# Deformation Behavior, a Flow Stress Model Considering the Contribution of Strain and Processing Maps in the Isothermal Compression of a Near-α Ti–3.3Al–1.5Zr–1.2Mo–0.6Ni Titanium Alloy

**DOI:** 10.3390/ma15093346

**Published:** 2022-05-06

**Authors:** Weixin Yu, Junhui Cao, Shusen Hou, Guanglong Wang, Yue Li, Shaoting Lang

**Affiliations:** School of Mechanical and Electrical Engineering, Xinxiang University, Xinxiang 453003, China; caojunhui@me.com (J.C.); shusen.hou@xxu.edu.cn (S.H.); wangglo@163.com (G.W.); liyue@xxu.edu.cn (Y.L.); shaotinglang@xxu.edu.cn (S.L.)

**Keywords:** titanium alloy, compression tests, deformation behavior, model, processing map

## Abstract

In the present study, isothermal compression tests are conducted for a near-α Ti–3.3Al–1.5Zr–1.2Mo–0.6Ni titanium alloy at deformation temperatures ranging from 1073 K to 1293 K and strain rates ranging from 0.01 s^−1^ to 10 s^−1^ on a Gleeble-3500 thermomechanical compressor. The results show that, in the initial stage of the compression, the flow stress rapidly increases to a peak value because of elastic deformation, and then the alloy enters the plastic deformation stage and the flow stress slowly decreases with the increase in strain and tends to gradually stabilize. In the plastic deformation stage, the flow stress significantly decreases with the increase in the deformation temperature and the decrease in strain rate. A flow stress model considering the contribution of the strain is established, and the relative error between the calculated and the experimental values is 3.72%. The flow stress model has higher precision and can efficiently predict the flow behavior in the isothermal compression of the alloy. Furthermore, the processing map of the Ti–3.3Al–1.5Zr–1.2Mo–0.6Ni alloy is drawn. Based on the processing map, the influence of process parameters on power dissipation efficiency and stability parameters is analyzed, and the optimized hot working process parameters are pointed out.

## 1. Introduction

Because of its excellent properties, titanium alloy has more and more applications in many fields [1,2]. In the Ti–3.3Al–1.5Zr–1.2Mo–0.6Ni alloy, Al and Zr are used to strengthen the α phase, and Mo and Ni are used to strengthen the β phase. It is a medium-strength titanium alloy with a tensile ultimate strength of 700 MPa. Since there is no precipitated compound phase in the solidified structure of the Ti–3.3Al–1.5Zr–1.2Mo–0.6Ni alloy, because of the lower content of alloying elements, the alloy has a good welding performance and a broad application prospect in ocean engineering. The plastic forming process is commonly used in manufacturing titanium alloy components. However, the near-α titanium alloy has few slip systems, poor plasticity, and poor plastic forming processability [3,4,5]. Therefore, it is particularly important to establish a flow stress model for the numerical simulation prediction of the hot forming of the Ti–3.3Al–1.5Zr–1.2Mo–0.6Ni titanium alloy.

The flow stress model reflects the dynamic response of the flow stress to the deformation process parameters during the thermoplastic processing of the material. Accurately describing this model is the basis of metal plastic deformation process design and control, and it is also the prerequisite for the numerical simulation of the metal plastic forming process [6,7,8,9].

To date, a number of models predicting the flow stress in the deformation process of various titanium alloys have been reported in the literature. Yu et al. [10] applied four different models for TG6 titanium alloy, and the Arrhenius model and modified Johnson–Cook model were more accurate than the other two models. Hajari et al. [11] found that the strain-compensated Arrhenius model provided a more accurate prediction of the flow behavior of the Ti–6242S alloys. Cui et al. [12] proposed a constitutive model for TC11 alloy based on the Arrhenius-type hyperbolic sine method. Zeng et al. [13] proposed a constitutive model for pure titanium on the basis of an Arrhenius equation. Various artificial neural networks are also widely used in flow stress models, but their accuracy is based on a large number of experiments [14,15,16,17,18]. According to the literature, Arrhenius equations are suitable for the construction of a flow stress model of a titanium alloy. However, the original Arrhenius model does not consider the effect of strain, which can not be ignored in the hot working process of titanium alloys.

The processing map is a superposition of the power-dissipation efficiency map and instable map established based on the dynamic material model. In the power-dissipation efficiency map, the power-dissipation efficiency is a function of the deformation process parameters, while the instable map can depict the areas in which steady and unsteady flows occur [19,20]. The processing map not only describes the deformation mechanism of a specific microstructure in the definite area, but also depicts the instable flow area that should be avoided, and the deformation process parameters can be optimized [21,22,23,24,25]. It is of great significance to establish the processing map for determining the processing parameters of new materials.

In this paper, isothermal compressions of the Ti–3.3Al–1.5Zr–1.2Mo–0.6Ni alloy are carried out. Based on the compression experimental data, the deformation behavior of the alloy is studied, and a flow stress model considering the contribution of strain is established, and finally the processing map is used to optimize the deformation process parameters in the isothermal compression of the Ti–3.3Al–1.5Zr–1.2Mo–0.6Ni alloy.

## 2. Materials and Methods

**Figure 1:**
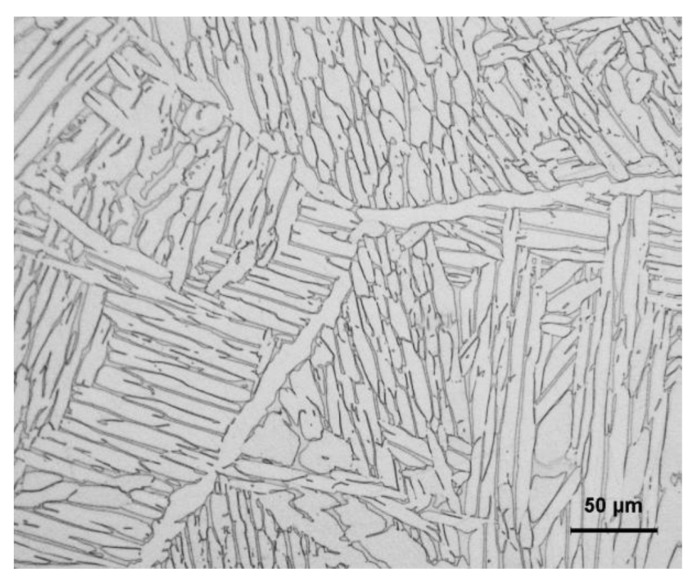
The microstructure of the Ti–3.3Al–1.5Zr–1.2Mo–0.6Ni alloy.

The material used in this paper was a Ti–3.3Al–1.5Zr–1.2Mo–0.6Ni ingot. The manufacturing process of the ingot was as follows: sponge Ti, sponge Zr, Ti–Mo master alloy, and Ti–Ni master alloy were mixed according to the required proportion and pressed into a consumable electrode, which was melted in a vacuum consumable arc furnace 3 times to obtain a titanium alloy ingot with good uniformity. The measured chemical composition (in wt%) of the Ti–3.3Al–1.5Zr–1.2Mo–0.6Ni alloy was 3.26 Al, 1.46Zr, 1.22Mo, and 0.58Ni, and the contents of impurity elements, such as C, Si, H, N, and O, were less than 0.01, 0.01, 0.001, 0,01, and 0.10, respectively, and balance Ti. The microstructure of the ingot is shown in Figure 1. The β-transus temperature for this alloy was 940 °C (1213 K), determined by the differential thermal analysis on a TGA/DSC1 synchronous thermal analyzer.

**Figure 2:**
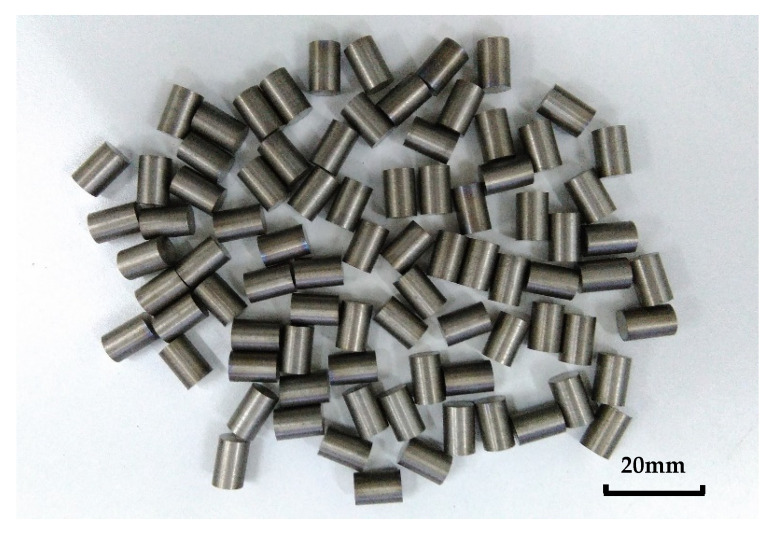
The thermal simulation compression specimens.

**Figure 3:**
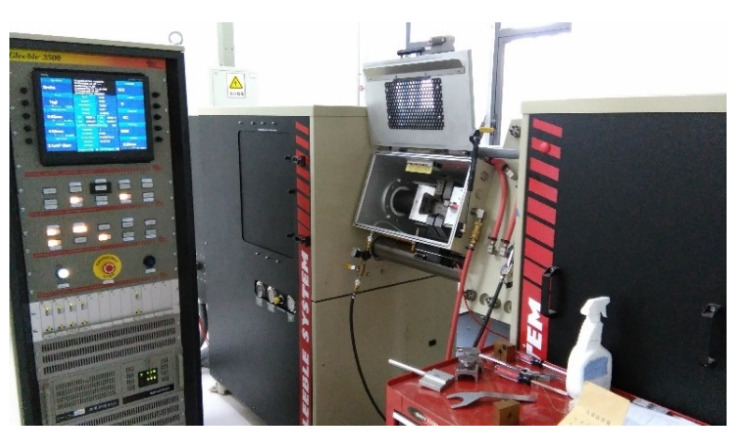
The thermomechanical compressor equipment.

Cylindrical specimens were used for thermal compression experiments, as shown in Figure 2. The diameter of the specimens was 8 mm and the height was 12 mm. The isothermal compressions were conducted in a GLEEBLE-3500 simulator as shown in Figure 3. In this study, the compression parameters used included the deformation temperatures of 1073 K, 1123 K, 1153 K, 1183 K, 1213 K, 1243 K, and 1293 K; the strain rates of 0.01 s^−1^, 0.1 s^−1^, 1.0 s^−1^, and 10.0 s^−1^; and a height reduction of 60%. The specimens were held at the deformation temperature for 5 min to ensure thermal equilibrium before starting the compression. Compression experiments were carried out under the preset process parameters, and the flow stress data of the materials were automatically recorded during the compression.

## 3. Results and Discussion

### 3.1. Deformation Behavior

**Figure 4:**
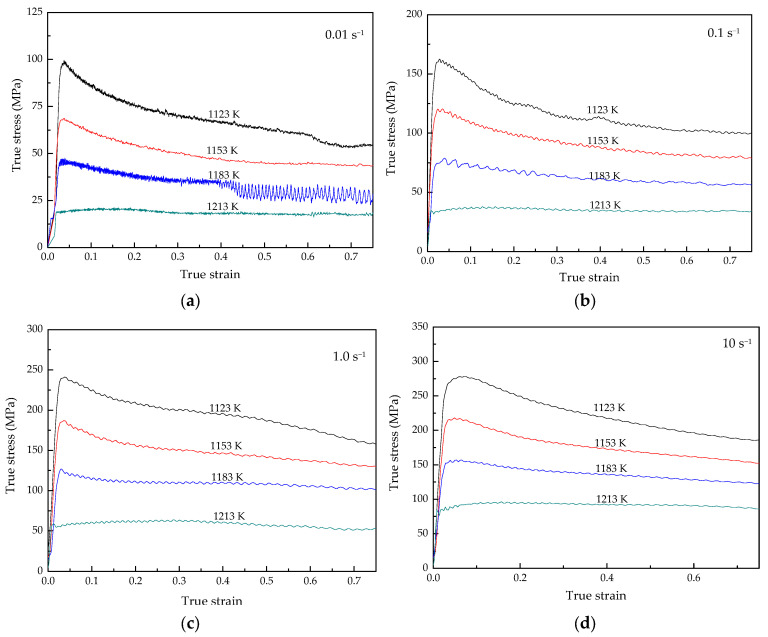
Flow stress–strain curves of Ti–3.3Al–1.5Zr–1.2Mo–0.6Ni titanium alloy at different strain rates: (**a**) 0.01 s^−1^, (**b**) 0.1 s^−1^, (**c**) 1.0 s^−1^, and (**d**) 10 s^−1^.

Flow stress–strain curves of the Ti–3.3Al–1.5Zr–1.2Mo–0.6Ni titanium alloy at different deformation parameters are shown in Figure 4. It can be seen from Figure 4 that the flow stresses rapidly increase with an increasing strain at the initial stage of deformation, and reach a peak value. After the peak stress value, the flow stress slowly decreases and tends to gradually stabilize. In other words, the Ti–3.3Al–1.5Zr–1.2Mo–0.6Ni titanium alloy shows typical elastic-plastic characteristics. In the initial stage of the experiment (elastic deformation is dominant), the flow stress increment is proportional to the strain increment. When the stress reaches the yield strength, the slope of the curve decreases significantly, and the material enters the plastic deformation stage. The slope of the true stress–strain curves in the elastic stage is the elastic modulus of the Ti–3.3Al–1.5Zr–1.2Mo–0.6Ni alloy. In the plastic deformation stage, the softening mechanism is strengthened, and the flow stress gradually decreases until it reaches the steady-state flow stage. It is generally considered that the softening mechanism during the hot deformation of the titanium alloy is mainly dynamic recovery because of its high stacking-fault energy, which is conducive to the dislocation of the cross slip [26,27,28,29].

**Figure 5:**
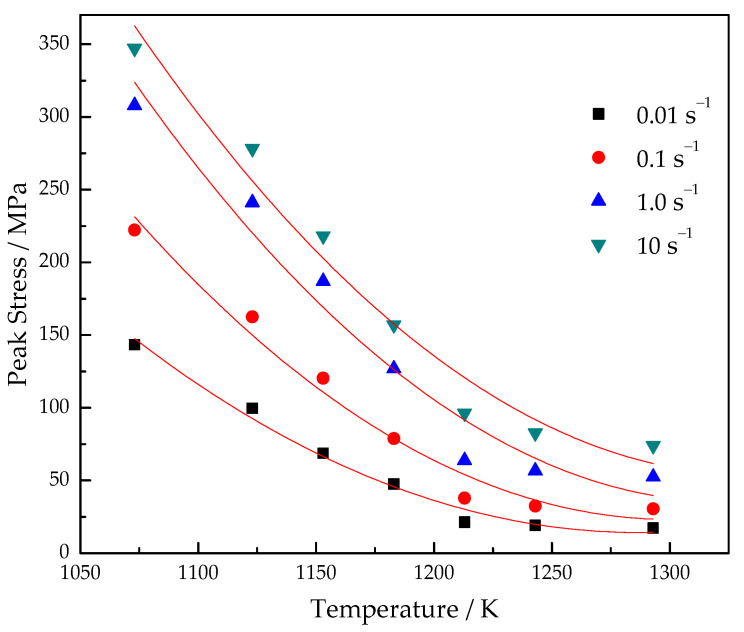
Peak stress of Ti–3.3Al–1.5Zr–1.2Mo–0.6Ni titanium alloy at different deformation parameters.

The flow stress obviously increases with the increase in the strain rate, as shown in Figure 4. This is mainly because the elastic deformation degree increases with the increasing strain rate in the initial stage of the experiment. The peak stresses in the isothermal compression of the Ti–3.3Al–1.5Zr–1.2Mo–0.6Ni titanium alloy are shown in Figure 5. When the deformation temperature is 1123 K and the strain rate increases from 0.01 s^−1^ to 10.0 s^−1^, the peak flow stress correspondingly increases from 99 MPa to 278 MPa, whereas, at the deformation temperature of 1213 K, the peak flow stress increases from 22 MPa at 0.01 s^−1^ to 96 MPa at 10.0 s^−1^. This means that, at lower temperatures, the effect of strain rate on flow stress is more significant. This is because at lower temperatures, the dislocation slip is not easy to operate at the initial stage of the deformation, and the rapid deformation makes the elastic deformation account for a larger proportion.

The flow stress obviously decreases with increasing deformation temperature, as shown in Figure 4, which indicates that the deformation of the Ti–3.3Al–1.5Zr–1.2Mo–0.6Ni alloy is a thermal activation process. The thermal activation of materials increases with the increasing deformation temperature, which leads to the increase in softening caused by dynamic recovery [30,31,32]. Therefore, the flow stress of materials decreases accordingly. With the progress of plastic deformation, the hardening caused by plastic deformation and softening by dynamic recovery gradually reach a balance, and the metal enters a steady-flow stage. Additionally, at higher deformation temperatures, the alloy can quickly reach the hardening–softening equilibrium and enter the steady-flow stage.

### 3.2. Flow Stress Model

The flow stress during metal thermal deformation can be described by the following three Arrhenius-type relationships [10,11,12,13]:

(1) $$\dot{\varepsilon }\exp\left(\frac{Q}{RT}\right)=A\sigma^{n}$$

(2) $$\dot{\varepsilon }\exp\left(\frac{Q}{RT}\right)=A_{1}\exp(n_{1}\sigma )$$

(3) $$\dot{\varepsilon }\exp\left(\frac{Q}{RT}\right)=A_{2}\sinh{\left(\alpha \sigma \right)}^{n_{2}}$$

where *σ* is the flow stress (MPa); $\dot{\varepsilon }$ is the strain rate (s^−1^); *T* is the deformation temperature (K); *Q* is the activation energy of deformation (kJ/mol); *R* is the gas constant (8.3145 J·mol^−1^·K^−1^); *n*, *n*_1_, and *n*_2_ are the stress exponents; and *A*, *A*_1_, *A*_2_ and α are the constants related to the material.

By taking the natural logarithm, Equations (1)–(3) can be written as follows:

(4) $$\ln\sigma =\ln\dot{\varepsilon }/n+Q/(nRT)-\ln A/n$$

(5) $$\sigma =\ln\dot{\varepsilon }/n1+Q/(n1RT)-\ln A1/n1$$

(6) $$\ln[\sinh(\alpha \sigma )]=\ln\dot{\varepsilon }/n_{2}+Q/(n_{2}RT)-\ln A_{2}/n_{2}$$

**Figure 6:**
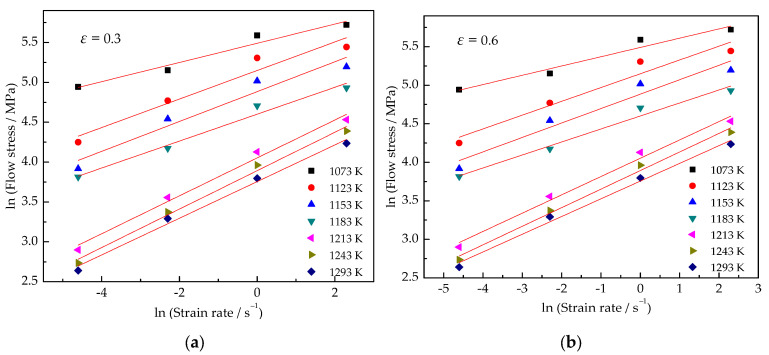
Relationship between ln (strain rate) and ln (flow stress) at strains of (**a**) 0.3 and (**b**) 0.6.

**Figure 7:**
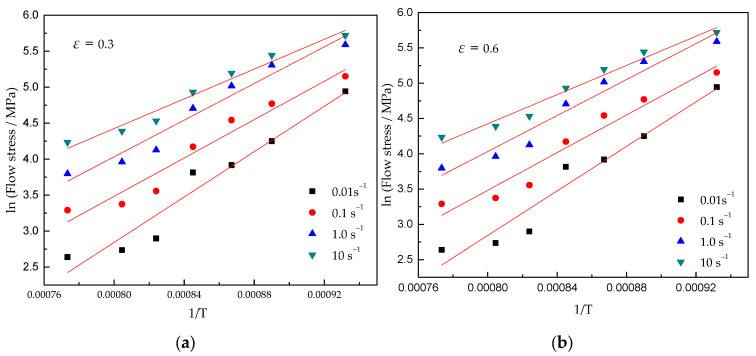
Relationship between 1/T and ln (flow stress) at strains of (**a**) 0.3 and (**b**) 0.6.

By plotting the experimental data, as shown in Figure 6 and Figure 7, it can be concluded that Equation (1) is suitable for the Ti–3.3Al–1.5Zr–1.2Mo–0.6Ni alloy.

**Figure 8:**
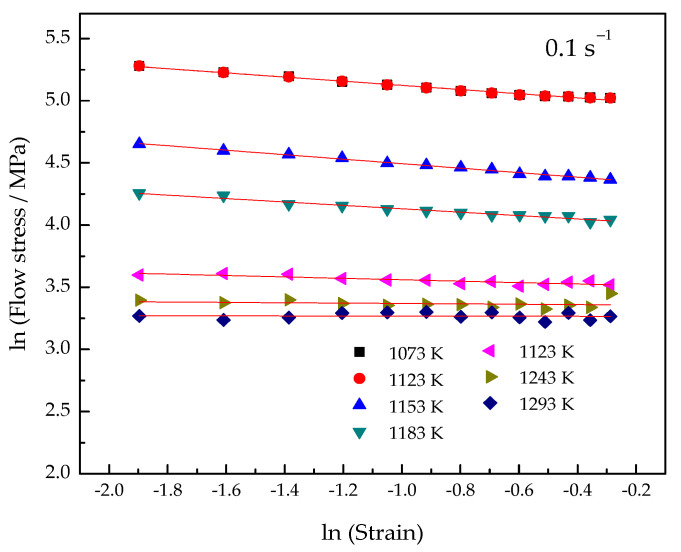
Relationship between ln (strain) and ln (flow stress).

There is no strain term in the Arrhenius equation. However, the strain has an obvious influence on the flow stress, as shown in Figure 4, so it is very necessary to show the strain term in the flow stress model. By plotting the experimental data, it is found that there is also a linear relationship between lnσ and lnε, as shown in Figure 8.

Considering the influence of strain, the flow stress is expressed by the following formula:

(7) $$\sigma =f_{1}\left(\dot{\varepsilon },T\right)\times f(\varepsilon )$$

As lnσ and lnε show a linear relationship according to Figure 8, the flow stress model of the Ti–3.3Al–1.5Zr–1.2Mo–0.6Ni titanium alloy can be expressed as follows:

(8) $$\varepsilon^{m}\dot{\varepsilon }\exp\left(\frac{Q}{RT}\right)=A_{2}\sigma^{n}$$

where *m* is a constant related to the material.

Take the logarithms of both sides of Equation (8):

(9) $$\ln\sigma =-\frac{\ln A_{2}}{n}+\frac{1}{n}\left(\ln\dot{\varepsilon }+\frac{Q}{RT}\right)+\frac{m}{n}\ln\varepsilon$$

Introducing the *Z*-*H* parameter, Equation (9) can be rewritten as follows:

(10) lnσ=A+BlnZ+Clnε

where $Z=\dot{\varepsilon }\exp\left(\frac{Q}{RT}\right)$, *A*, *B*, and *C* are the constants related to the material.

In order to accurately describe the deformation behavior, the improved flow stress model is as follows:

(11) $$\ln\sigma =B_{0}+B_{1}\ln Z+B_{2}{\left(\ln Z\right)}^{2}+B_{3}{\left(\ln Z\right)}^{3}+B_{4}\ln\varepsilon$$

where, *B*_0_, *B*_1_, *B*_2_, *B*_3_, and *B*_4_ are the material parameters to be determined.

To establish the flow stress model expressed by *Z*-*H* parameters, the deformation activation energy must be first determined.

Transform Equation (1) by taking the logarithm on both sides:

(12) $$\ln\dot{\varepsilon }=\ln A+n\ln\sigma -\frac{Q}{RT}$$

When the strain rate remains constant, take the partial derivatives on both sides of Equation (12) and transform it to

(13) $$Q=nR\frac{\partial \ln\sigma }{\partial (1/T)}|{}_{\dot{\varepsilon }}\approx nR\frac{\Delta \ln\sigma }{\Delta (1/T)}|{}_{\dot{\varepsilon }}$$

According to the thermodynamics of the irreversible process, when the deformation temperature and strain are constant, the following formula can be introduced:

(14) $$\sigma =C{\dot{\varepsilon }}^{m}|{}_{\varepsilon ,T}$$

where *C* is a constant and *m* is the strain rate sensitivity index.

Take the logarithms on both sides of Equation (14) and transform it to

(15) $$\ln\sigma =\ln C+m\ln\dot{\varepsilon }|{}_{\varepsilon ,T}$$

Take the partial derivative on both sides of Equation (15), and because of *n* = 1/*m*, it can be transformed to

(16) $$n=\frac{\partial \ln\dot{\varepsilon }}{\partial \ln\sigma }|{}_{\varepsilon ,T}\approx \frac{\Delta \ln\dot{\varepsilon }}{\Delta \ln\sigma }|{}_{\varepsilon ,T}$$

**Figure 9:**
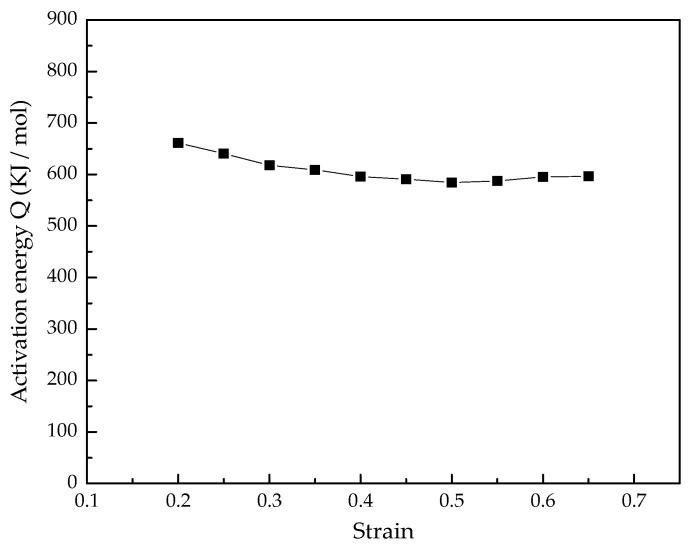
Activation energy of deformation of Ti–3.3Al–1.5Zr–1.2Mo–0.6Ni alloy.

The slopes of the $\ln\sigma -\ln\dot{\varepsilon }$ and lnσ-1/T curves are calculated from Figure 6 and Figure 7, respectively. The slope values are substituted into Equations (16) and (13), and the deformation activation energy of the Ti–3.3Al–1.5Zr–1.2Mo–0.6Ni alloy in *α* + *β* phase field are obtained, as shown in Figure 9. The average deformation activation energy is 603.6 KJ/mol^−1^.

After the deformation activation energy is determined, multiple regression analysis is performed on Equation (11), and each coefficient is shown in Table 1.

**Figure 10:**
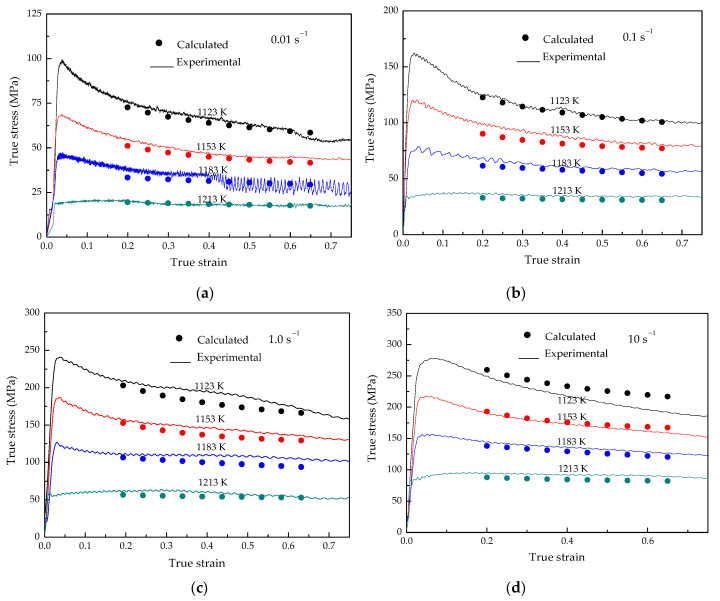
Comparison of the calculated with the experimental flow stress: (**a**) 0.01 s^−1^, (**b**) 0.1 s^−1^, (**c**) 1.0 s^−1^ and (**d**) 10 s^−1^.

The flow stress model is validated by the experimental data of the Ti–3.3Al–1.5Zr–1.2Mo–0.6Ni alloy. Figure 10 shows the comparison between the calculated results of the flow stress model established in this paper and the experimental data. From Figure 10, it can be observed that the flow stress calculated by the flow stress model can achieve satisfactory accuracy. The average relative error between the calculated values by the model and the experimental data is 3.72%. Therefore, the flow stress model of the Ti–3.3Al–1.5Zr–1.2Mo–0.6Ni alloy established in this paper is suitable to describe the flow behavior of the Ti–3.3Al–1.5Zr–1.2Mo–0.6Ni alloy during high-temperature deformation.

### 3.3. Processing Map

The processing map was established based on the dynamic material model (DMM) theory. According to the DMM, the energy input by external force to the workpiece (*P*) was used for the deformation and microstructure evolution. The energy dissipated due to plastic deformation can be represented by *G*, while the energy dissipated due to tissue evolution can be represented by *J*, which can be described in the following form [19,20]:

(17) $$P=\sigma \dot{\varepsilon }=\int_{0}^{\sigma }\dot{\varepsilon }d\sigma +\int_{0}^{\dot{\varepsilon }}\sigma d\dot{\varepsilon }=J+G$$

where *σ* is the flow stress (MPa) and $\dot{\varepsilon }$ is the strain rate (s^−1^).

The distribution ratio of *G* and *J* can be expressed by the following formula:

(18) $$\frac{dJ}{dG}=\frac{\dot{\varepsilon }d\sigma }{\sigma d\dot{\varepsilon }}=\frac{d(\log\sigma )}{d(\log\dot{\varepsilon })}=m$$

where *m* is the strain-rate sensitivity index.

*J* can be written as:

(19) $$J=\frac{m}{m+1}\times \sigma \times \dot{\varepsilon }$$

The power dissipation efficiency (*η*) is defined by

(20) $$\eta =\frac{J}{J_{max}}=\frac{2m}{m+1}$$

**Figure 11:**
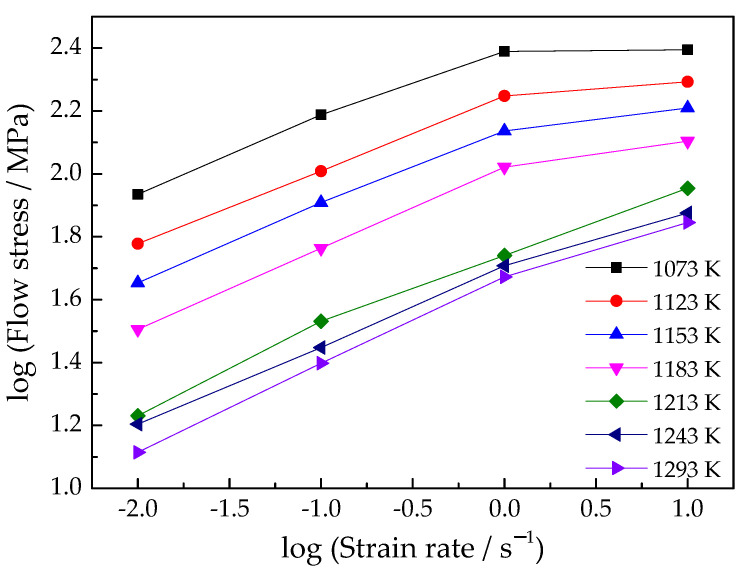
Relationship between log(strain rate) and log(flow stress).

Figure 11 shows the relationship between $\log\dot{\varepsilon }$ and logσ at the strain of 0.6. As can be observed from Figure 11, when the deformation temperature is above 1213 K, $\log\dot{\varepsilon }$ and logσ are approximately linear; whereas, when it is below 11,183 K, $\log\dot{\varepsilon }$ and logσ are nonlinear. Therefore, polynomial can be used to represent the logσ in the whole deformation temperature range, as shown in (21):

(21) $$\log\sigma =a+b\log\dot{\varepsilon }+c{\left(\log\dot{\varepsilon }\right)}^{2}+d{\left(\log\dot{\varepsilon }\right)}^{3}$$

Then, the strain rate sensitivity index *m* can be expressed as:

(22) $$m=\frac{d(\log\sigma )}{d(\log\dot{\varepsilon })}=b+2c\log\dot{\varepsilon }+3d{\left(\log\dot{\varepsilon }\right)}^{2}$$

**Figure 12:**
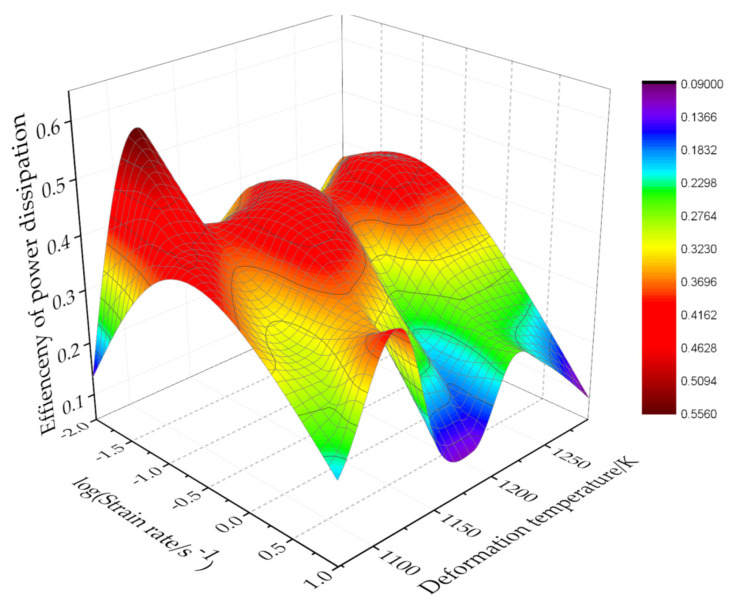
Map of the power dissipation efficiency of the isothermal compression of the Ti–3.3Al–1.5Zr–1.2Mo–0.6Ni alloy.

Substitute the logσ value of each point to obtain the *m* value of the corresponding point. Then, the power dissipation efficiency of the Ti–3.3Al–1.5Zr–1.2Mo–0.6Ni titanium alloy under different deformation temperature and strain rates is obtained by substituting value *m* into Equation (20). Through the interpolation method, the energy-dissipation diagram for all the whole experimental conditions can be obtained, as shown in Figure 12.

Based on Zeigler’s maximum entropy yield principle, Kumar proposed the following criteria for metallurgical instability in metal forming [19,20]:

(23) $$\xi (\dot{\varepsilon })=\frac{\partial \log\frac{m}{m+1}}{\partial \log\dot{\varepsilon }}+m<0$$

where $\xi (\dot{\varepsilon })$ is the stability parameter.

According to Equations (22) and (23):

(24) $$\xi (\dot{\varepsilon })=\frac{\partial \log\frac{m}{m+1}}{\partial \log\dot{\varepsilon }}+m=\frac{2c+6d(\log\dot{\varepsilon })}{m(m+1)\ln10}+m$$

**Figure 13:**
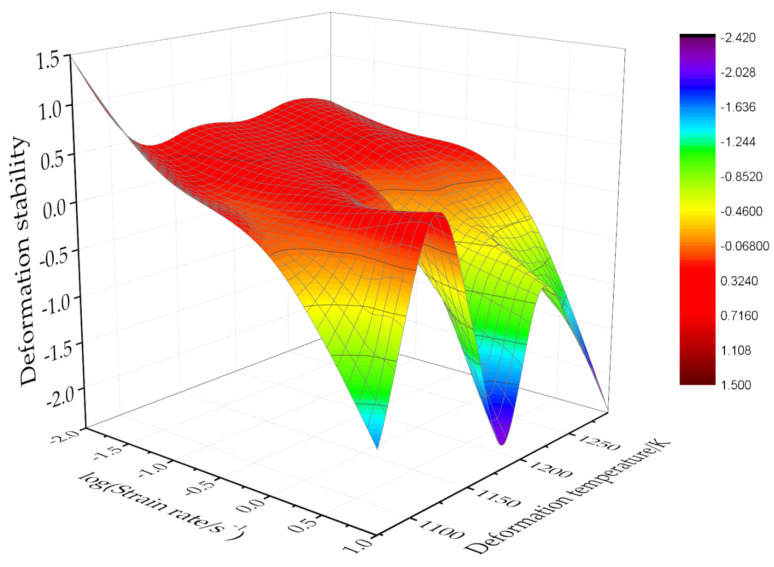
Instable map of the isothermal compression of the Ti–3.3Al–1.5Zr–1.2Mo–0.6Ni alloy.

**Figure 14:**
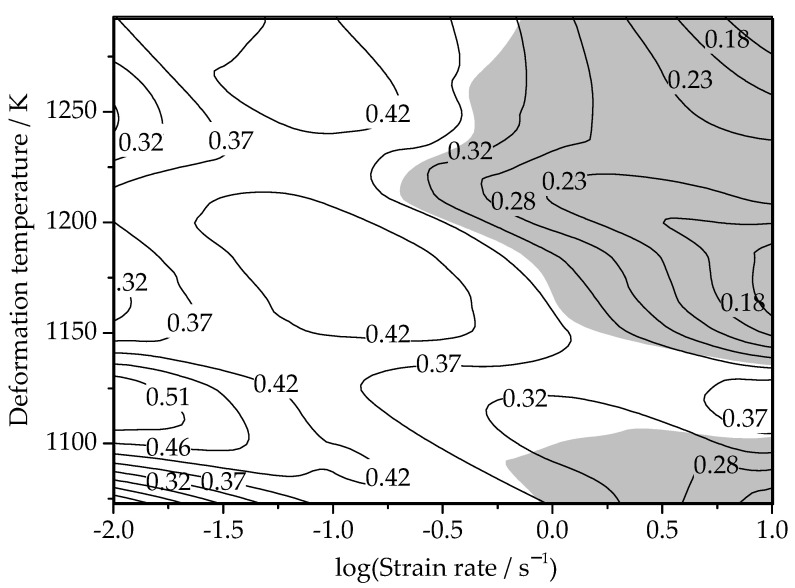
Processing map of the isothermal compression of the Ti–3.3Al–1.5Zr–1.2Mo–0.6Ni alloy.

Substituting the $\log\dot{\varepsilon }$ and *m* values of each point can obtain the $\xi (\dot{\varepsilon })$ value under different deformation temperatures and strain rates. The instable map was obtained by the interpolation method, as shown in Figure 13. The processing map of the Ti–3.3Al–1.5Zr–1.2Mo–0.6Ni titanium alloy at the strain of 0.6 can be obtained by superimposing the maps in Figure 12 and Figure 13, as shown in Figure 14. The contour value in the processing map is the power dissipation efficiency *η*, and the shaded part represents the instable deformation regions.

As can be observed from Figure 14, in the deformation temperature range of 1105–1130 K and strain rate range of 0.01–0.02 s^−1^, the power dissipation efficiency reaches the maximum value of 0.51. When deformed under this set of process parameters, more energy is used for microstructure evolution, while less energy is stored in the metal in the form of distortion energy, which is beneficial to the control of the metal microstructure.

Flow instability easily occurs at higher strain rates. From Figure 14, it can be observed that there are two instable deformation regions. The first is that the deformation temperature is 1123~1100 K and the strain rate is 0.6~10 s^−1^, and the second is 1130~1293 K and 0.5~10 s^−1^. When the Ti–3.3Al–1.5Zr–1.2Mo–0.6Ni titanium alloy is deformed in these instable deformation regions, various defects unfavorable to the microstructure may appear, so the hot working in these regions should be avoided. When the deformation temperature is 1105–1130 K, it is still a stable deformation region under the condition of high strain rates. Therefore, when high-speed equipment is used, such as a forging hammer, crank press, and friction press, the deformation temperature can only be set between 1105–1130 K.

## 4. Conclusions

(1) In the deformation temperature range of 1073 K to 1293 K and strain rate range of 0.01 s^−1^ to 10 s^−1^, the flow stress of the Ti–3.3Al–1.5Zr–1.2Mo–0.6Ni alloy was sensitive to deformation parameters. With the increase in the deformation temperature and the decrease in the strain rate, the flow stress of the alloy decreased significantly.

(2) With the increase in the strain, the flow stress of the Ti–3.3Al–1.5Zr–1.2Mo–0.6Ni alloy first rapidly increased and reached the peak. After the peak value, the flow stress slowly decreased with the increase in the strain and tended to gradually stabilize.

(3) Based on the experimental results of the thermal compression of the Ti–3.3Al–1.5Zr–1.2Mo–0.6Ni alloy, a flow stress model reflecting the influence of strain was established. The verification results of the flow stress model show that the average error between the calculated values of the model and the experimental values is 3.72%, which is suitable to describe the flow behavior of the Ti–3.3Al–1.5Zr–1.2Mo–0.6Ni alloy during high-temperature deformation.

(4) The processing map of the Ti–3.3Al–1.5Zr–1.2Mo–0.6Ni alloy was drawn. Based on the processing map, the effect of the deformation parameters on the power dissipation efficiency and stability parameter were analyzed, and the optimized parameters were the deformation temperatures of 1105–1130 K and strain rates of 0.01–0.02 s^−1^.

## Figures and Tables

**Table 1:** Material constants of Ti–3.3Al–1.5Zr–1.2Mo–0.6Ni titanium alloy in Equation (11).

$B_{0}$	$B_{1}$	$B_{2}$	$B_{3}$	$B_{4}$
3.94331	0.21924	−0.01144	−9.02243 × 10^−4^	−0.05766

## Data Availability

The data presented in this study are available on request from the corresponding author.
